# A Text Book of Practical Anatomy

**Published:** 1855-02

**Authors:** 


ART. VII.—*A Text-Book of Practical Anatomy.* By ROBERT HARRISON, M. D., M. R. I. A., Fellow of the Royal Colleges of Surgeons of Ireland and of England, Prof. of Anatomy and Surgery of the University of Dublin, etc. With additions by an American Physician. Second Edition. New York: S. S. and W. Wood. 1855.

*The Dublin Dissector or Manual of Anatomy, etc.* By ROBERT HARRISON, A. M., M. B., T. C. D., etc. Third American, from the fifth and enlarged Dublin Edition. With additions by Robert Watts, Jr., M. D., Prof. of Anat. in Coll. Phys. and Surgeons, N. Y. New York: Samuel S. and Wm. Wood. 1854.

The Dublin Dissector has enjoyed an immense sale, and has long been the favorite text-book in the dissection room. Its descriptions are clear and accurate, and its instructions to the dissector are such as to make the most advantageous use of material. Its wide spread popularity among students is no small recommendation of its merits.

"Harrison's Anatomy" is the Dublin Dissector in octavo, with plates, and some additions from the competent pen of Prof. Watts. It must share in the popularity of its less pretentious original, and may safely be commended to the student of descriptive anatomy. H.

